# Heveenoid.—The Rubber of the Future

**Published:** 1881-03

**Authors:** Hy. A. Mott


### ARTICLE VI.

### *Heveenoid—The Rubber of the Future.*

We condense the following from a pamphlet written by Prof. Henry A. Mott, Jr., and published by Trow's Printing and Book-binding Company, of 201–203 East 12th street, New York:

Heveenoid is the name of a new product which is destined to supplant the soft and hard vulcanized India rubber which has for so long a time supplied the market. The base of Heveenoid is India rubber, whence the name, from *heeven*, the name given by the natives of South America to the milky juice of the India rubber tree; and *oid*, a Greek termination signifying *like*. The combination of this base with camphor vulcanized by sulphur constitutes *Heveenoid*. The discovery of Goodyear and Day, that sulphur will vulcanize India rubber, first made this substance of value in the industrial arts. While this discovery must always be looked upon as one of great value, the discovery of the new product, Heveenoid, by Henry Gerner, opens a new era in the rubber industry, and will, unquestionably, to a very great extent, in time, take the place of the vulcanized rubber of to-day.

The proportions of the constituents to make a soft and hard Heveenoid may be approximately given as follows:

| | | |
|---|---|---|
| Rubber | 2 parts. | 3 parts. |
| Camphor | 2 " | 2 " |
| Lime | 1-16 " | —— |
| Glycerine | —— | ½ " |
| Sulphur | ¼ " | 8 " |

Para is best for hard Heveenoid, while Nicaragua rubber answers very well for soft Heveenoid, and, in fact, is somewhat more adapted.

It is a well known fact that sulphur will only combine to a very limited extent with rubber, only about one to three per cent. entering into chemical combination, the remainder of the sulphur added existing only in mechanical suspension. This fact is proved by two simple experiments —*first*, one or two per cent. of sulphur is sufficient to vulcanize with, and produce the change; and *second*, if vulcanized rubber is ignited, instead of burning with a steady, smoky flame, it throws off sparks of ignited sulphur, and if the flame be extinguished, the sulphur will be seen to solidify. The odor also of sulphurous acid is very marked. This is not the case with Heveenoid; all the constituents of this new product are chemically united. The sulphur unites with the camphor, forming a sulphide of camphene, which either dissolves or is dissolved by the rubber; it seems, however, more probable that the former is the case.

The temperature at which the constituents unite chemically is just above the melting point of camphor. In the manufacture of ordinary rubber, if too much sulphur is added the product is hard and brittle. If such rubber be placed and left sufficiently long in molten camphor it swells up, and combines with the camphor, acquiring toughness and flexibility, and becomes a desirable material; in other words, it becomes Heveenoid.

The process of manufacturing Heveenoid, is, of course, patented, the patent being held by the Heveenoid Manufacturing Co., of New York. At Hoboken, N. J., works are erected sufficiently large to make over three thousand pounds of stock a day. This Company, however, only manufactures specialties, and sells licenses to other companies. The great consideration in favor of Heveenoid is the fact that it can be manufactured, and put on the market for about thirty to fifty per cent. cheaper than the ordinary vulcanized rubber.

The properties of good vulcanized rubber are: Elasticity, pliability, durability, insolubility, unalterability by climate, or artificial heat or cold, inadhesiveness, impermeability to air, gases, and liquids, odor, plasticity, facility of receiving every style of printing, facility of being ornamented by painting, bronzing, gilding, japaning, and mixing with colors, non-electric quality, spreading quality.

A thorough investigation into the merits of Heveenoid, which I have had occasion to make, developed the fact that Heveenoid not only possesses the above mentioned properties of vulcanized India rubber, but in many instances possesses them in an infinitely superior degree. As for example, Heveenoid is far more pliable, durable, and insoluble than ordinary India rubber. Being a chemical combination is less impermeable to air, gases and liquids. As regards odor, it is far superior to vulcanized rubber, which, as is well known, possesses a very disagreeable, sulphur odor. Soft Heveenoid smells of camphor, which renders it of great service as a moth and insect destroyer. Its application for lining of closets or wrapping of furs, etc., as also for submarine cables opens a large field for it. Hard Heveenoid, when rubbed or when warm, has a slight odor of camphor, but it is not noticed unless under these conditions—the odor being pleasant, is much to be preferred to the old sulphur odor. Heveenoid possesses a great value over rubber in the manufacture of jewelry, as the sulphur, being in chemical combination, permits of the use of poor as we.l as fine gold in ornamenting it. None but eighteen carat gold can be used with the ordinary vulcanized rubber, otherwise, owing to the free sulphur in its composition, the gold would change color. As regards coloring, Heveenoid, is far superior to rubber, as it may be colored any desired tint. This is something which the manufacturers of rubber have often attempted to do, but have utterly failed.

Another property Heveenoid possesses is the facility with which it can be sawed and designed. A saw may be used with Heveenoid for from one-half to three-quarters of a day without being sharpened. The sharpening of saws in the working of ordinary vulcanized India rubber is a matter of great expense, as they must be sharpened about every half hour. It is the friction of the saws against the uncombined sulphur in India rubber which dulls them; the sulphur in Heveenoid being in chemical combination, can offer no such frictional resistance.

The discovery of Heveenoid will certainly give rubber and camphor a much wider application in the arts, as this new product is adapted for many new purposes, where ordinary India rubber would be useless. It seems strange that the combinations of gum camphor with rubber had not been thought of before, especially when the fact is considered that Goodyear obtained his first patent in 1844, nearly forty years ago. The only way it can be accounted for is the fact that the India rubber business has been a monopoly, and like most monopolies, original investigation in the line of improvements have been prosecuted only to a limited extent. The merits of Heveenoid are such as to indicate an exceptionably profitable future, both as a benefit to the industrial arts and as a business of itself.—*Odontographic Journal.*

